# CT radiomics to predict Deauville score 4 positive and negative Hodgkin lymphoma manifestations

**DOI:** 10.1038/s41598-022-24227-0

**Published:** 2022-11-21

**Authors:** Laura J. Jensen, Julian M. M. Rogasch, Damon Kim, Juliana Rießelmann, Christian Furth, Holger Amthauer, Bernd Hamm, Ingo G. Steffen, Thomas Elgeti, Sebastian N. Nagel

**Affiliations:** 1 Department of Radiology, Charité-Universitätsmedizin Berlin, Corporate Member of Freie Universität Berlin, Humboldt-Universität zu Berlin, and Berlin Institute of Health, Hindenburgdamm 30, 12203 Berlin, Germany; 2 Department of Nuclear Medicine, Charité-Universitätsmedizin Berlin, Corporate Member of Freie Universität Berlin, Humboldt-Universität zu Berlin, and Berlin Institute of Health, Augustenburger Platz 1, 13353 Berlin, Germany; 3 Berlin Institute of Health (BIH), Berlin, Germany

**Keywords:** Haematological cancer, Oncology, Diagnostic markers, Predictive markers

## Abstract

18F-FDG-PET/CT is standard to assess response in Hodgkin lymphoma by quantifying metabolic activity with the Deauville score. PET/CT, however, is time-consuming, cost-extensive, linked to high radiation and has a low availability. As an alternative, we investigated radiomics from non-contrast-enhanced computed tomography (NECT) scans. 75 PET/CT examinations of 43 patients on two different scanners were included. Target lesions were classified as Deauville score 4 positive (DS4+) or negative (DS4−) based on their SUVpeak and then segmented in NECT images. From these segmentations, 107 features were extracted with PyRadiomics. All further statistical analyses were then performed scanner-wise: differences between DS4+ and DS4− manifestations were assessed with the Mann–Whitney-U-test and single feature performances with the ROC-analysis. To further verify the reliability of the results, the number of features was reduced using different techniques. The feature median showed a high sensitivity for DS4+ manifestations on both scanners (scanner A: 0.91, scanner B: 0.85). It furthermore was the only feature that remained in both datasets after applying different feature reduction techniques. The feature median from NECT concordantly has a high sensitivity for DS4+ Hodgkin manifestations on two different scanners and thus could provide a surrogate for increased metabolic activity in PET/CT.

## Introduction

^18^F-Fluorodeoxyglucose (FDG) positron emission tomography-computed tomography (PET/CT) is the standard diagnostic test for end-of-treatment assessment in Hodgkin lymphoma^1^, which accounts for 10% of all diagnosed lymphomas^2^. The metabolic activity in PET/CT is usually assessed with the Deauville Score (DS), a 5-point system to categorize FDG avidity. Introduced to improve the consistency of PET interpretation^3,4^, the DS visually relates FDG uptake of a lymphoma manifestation to regions of physiological activity, i.e., the liver and the mediastinal blood pool^5^. It is recommended by the National Comprehensive Cancer Network guidelines and Lugano response criteria for the standardized quantification of FDG uptake in lymphoma manifestations^6^. A DS of 3 or less is considered an adequate, and a DS of 4 or greater is considered an inadequate treatment response. This cutoff is of the highest relevance^7,8^.

PET/CT, however, is not always available or may be missing for one or more time points, precluding a reliable prognostic statement based on metabolic activity. It is also an expensive and time-consuming procedure, resulting in a high radiation dose for the often young patients^9^. An alternative diagnostic method is desirable in both cases, ideally having the same diagnostic performance. Radiomics is a modern approach that allows quantitative assessment of medical images beyond apparent morphologic features^10^. Features are extracted from a region of interest (ROI) by mathematical-statistical processing, resulting in many quantifiable features to characterize different image properties^10^. Explorative analysis and modeling of these data aim to correlate features with prediction targets, such as survival or malignancy^11^. For example, Mayerhoefer et al. were able to differentiate Glioblastoma from CNS Lymphoma with MR-derived radiomics and predict the survival of lymphoma patients with radiomics from ^18^F-FDG-PET data^12^. Or Milgrom et al., who estimated the relapse rate of mediastinal Hodgkin Lymphoma by building a model with the five most predictive radiomic features from baseline PET scans, yielding promising AUCs^13^.

Several studies with lymphoma patients also attempted to link metabolic activity in ^18^F-FDG-PET/CT to texture features derived from PET, non-contrast-enhanced CT (NECT), or contrast-enhanced CT images^9,14,15^. For example, Ganeshan et al. suggested a link between the non-contrast-enhanced CT-derived texture parameter kurtosis and shorter progression-free survival of lymphoma patients in an ^18^F-FDG-PET/CT study, including patients with Hodgkin lymphoma and aggressive Non-Hodgkin lymphoma^14^. In another study, Knogler et al. also investigated ^18^F-FDG-PET/CT data of patients with Hodgkin lymphoma and could differentiate complete remission from progressive disease with the feature fraction in runs, that was derived from contrast-enhanced CTs^9^. However, a clear tendency towards one texture feature or feature class that distinctly correlates with increased FDG uptake has not emerged yet. This may also be related to a known drawback regarding radiomics, which is their lack of reproducibility, particularly between different scanners^16–18^.

Therefore, this study aimed to explore if radiomic features from NECT images are linked to the metabolic activity of Hodgkin lymphoma manifestations and can discriminate between DS4-negative (DS 1–3) and DS4-positive (DS4 and DS5) manifestations. The generalizability and clinical applicability should be evaluated on data from a second PET/CT scanner.

## Materials and methods

### Study population and definition of target lesion

We included a total of 75 PET/CT datasets acquired in 43 patients. PET/CT scans were performed between September 2015 and March 2019. Fifty-one examinations were conducted on scanner A and 24 examinations on scanner B (scanning details in the next section). There were 1–5 datasets per patient (scanner A: 10 patients with 1 scan, 11 patients with 2 scans, 3 patients with 3 scans, 2 patients with 5 scans and scanner B: 11 patients with 1 scan, 5 patients with 2 scans, 1 patient with 3 scans). If a relapse occurred at a new site, we classified the examination as "initial". Overall, 26 initial and 49 interim examinations were included. Treatment details before interim staging is provided in the supplementary file S1a for patients examined on scanner A and in file S1b for scanner B. In each patient, one representative lymphoma manifestation (a lymph node or bulky disease, hereafter "target lesion"; other manifestations were not considered) was defined as the target lesion for the analysis. Usually, the target lesion was the one with the visually highest DS. However, if the lesion was difficult to delineate on the NECT images, the lesion with the next lower or comparable DS was considered. The volume of the lesion further had to be at least 1 cm^3^.

Details of the patients are summarized in Table 1. Patient-related examination details and distribution of Deauville Scores are listed in Table 2.

**Table 1:** Details of the patient population.

	Scanner A	Scanner B
Underlying disease	Hodgkin’s disease (all patients)	
Number of patients	26 (100%)	17 (100%)
Median age (years)	23 (IQR: 16–39)	36.5(IQR: – 22.25 to 52.25)
Sex	15 (58%) female, 11 (42%) male	7 (41%) female, 10 (59%) male
Median weight (kg)	64.5 (IQR: 55–92.5)	74 (IQR: 62–92)
Relapse (different site)*	2 patients (8%)	3 patients (18%)

Details of the included patients. Percentages of the patients are listed per scanner and may not total 100 due to rounding.

*IQR* interquartile range.

*Patients with relapse at a different site (if a relapse occurred at a new site, we classified the examination as "initial").

**Table 2:** Patient-related examination details and distribution of Deauville Scores.

	Scanner A		Scanner B	
Number of scans	51 (100%)		24 (100%)	
Median scans per patient	2 (IQR: 1–2)		1 (IQR: 1–2)	
Median blood sugar (mg/dl) prior to PET	92 (IQR: 81–100)		94 (IQR: 85–103)	
Median applied acitivity of F-18-FDG (MBq)	247.5 (IQR: 196.5–258.75)		255 (IQR: 205–264.5)	
Median uptake time (min)	68 (IQR: 64.75–81)		65 (IQR: 62–70)	

	DS4-positive	DS4-negative	DS4-positive	DS4-negative
Total	23 (45%)	28 (55%)	13 (54%)	11 (46%)
Initial scans	15 (29%)	1 (2%)	10 (42%)	0 (0%)
Interim scans	8 (16%)	27 (53%)	3 (13%)	11 (46%)

Target lesion Deauville score (DS)				
DS 1	0 (0%)		0 (0%)	
DS 2	25 (49%)		10 (42%)	
DS 3	3 (6%)		1 (4%)	
DS 4	4 (8%)		5 (21%)	
DS 5	19 (37%)		8 (33%)	
Median SUV_max_	3.58 (IQR: 1.89–7.62)		6.07 (IQR: 2.32–10.23)	
Median SUV_peak_	2.63 (IQR: 1.62–5.88)		3.65 (IQR: 1.84–6.68)	
Median size (mm^3^)	3093.8 (IQR: 869.4–6873.6)		4218.1 (IQR: 1516.4–8390.4)	

Details of the patient-related examination details and the distribution of the Deauville Scores. Percentages of the scans are listed scanner-wise and may not total 100 due to rounding.

*IQR* interquartile range.

### Image acquisition

FDG-PET/CT images were acquired on two different scanners: Scanner A (Gemini TF 16; Philips Medical Systems, Hamburg, Germany)^19^ and scanner B (Discovery MI; GE Medical Systems, Chicago, USA)^20^. All Patients fasted for ≥ 6 h before ^18^F-FDG injection and a blood glucose level of < 190 mg/dl was ensured. A median activity of 250 MBq ^18^F-FDG (interquartile range (IQR), 233–262 MBq) was administered intravenously. PET scan followed after a median uptake time of 68 min (IQR, 63.5–76.5 min). PET data were acquired from the skull base to the proximal femora in 3D acquisition mode (acquisition time, 2–3 min per bed position). PET raw data from scanner A were reconstructed using 3D ordered subset expectation maximization (OSEM) with a time of flight analysis (BLOB-OS-TF; iterations, 3; subsets, 33; filter, ‘smooth’). PET data from scanner B were reconstructed iteratively with Bayesian penalized likelihood reconstruction (GE “Q.Clear”) with a penalization factor β of 450, which included time of flight analysis and point spread function modeling^21^. Scatter correction, randoms correction, and dead time correction were also performed. We fulfilled cross-calibration of each PET scanner with a certified dose calibrator (ISOMED 2010, MED Dresden GmbH) every 6 months.

PET/CT scanning and reconstruction details are summarized in Table 3.

**Table 3:** Scanner and PET/CT scanning details.

	Scanner A	Scanner B
PET/CT model name	Gemini TF 16	Discovery MI
Manufacturer	Philips medical systems	GE medical systems
CT detector rows	16	64
kVp	120	120
mAs (automated tube current modulation)	50–100	50–100
Gantry rotation time (s)	0.5	0.5
CT matrix	512 × 512	512 × 512
CT field of view (mm)	436 × 436–688 × 688	500 × 500–700 × 700
Pixel spacing	0.8515625/0.8515625–1.34375/1.34375	0.9765625/0.9765625–1.367188/1.367188
Spacing between CT slices	− 1.5	− 2.78
Slice thickness (mm)	3.00	3.75
CT kernel	Body	Body
Patient position	Supine, head first	Supine, head first
PET Scintillator material	Lutetium–Yttrium Oxyorthosilicate (LYSO)	LYSO
PET photomultiplier technology	Conventional photomultiplier tubes	Silicon photomultipliers (SiPM)
PET time of flight capability	Yes	Yes
PET reconstruction	BLOB-OS-TF; iterations, 3; subsets, 33; filter, ‘smooth’	“Q.Clear” with penalization factor β of 450

CT scanning parameters used for examinations on the two different PET/CT-scanners included in the study.

*kVp* peak kilovoltage, *mAs* milliampere-seconds.

### Image analysis

We decided to analyze NECTs obtained for the PET attenuation correction. Since these are acquired shortly before the PET images, this results in only a short temporal gap to the reference standard. Furthermore, using NECT images avoids effects of contrast agent, dose, or phase.

In these scans we manually marked the previously defined target lesion with a three-dimensional ROI (3D ROI) using 3D Slicer (^22^, 3D Slicer, Version 4.10.0, http://www.slicer.org). The evaluation was carried out independently by two readers [Reader 1: S.N.N., Reader 2: L.J.J.] (reader 1: board-certified radiologist with over nine years of experience, reader 2: radiology resident with more than four years of experience). The readers were required to delineate as much of the target lesion as possible while keeping a minimum distance of 1–2 pixels from the edge.

**Figure 1:**
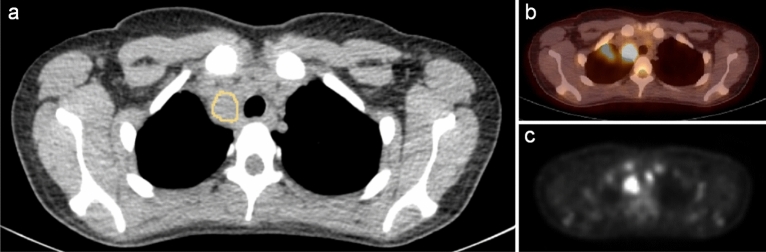
Illustration of ROI placement. (**a**–**c**) A 20-year-old female patient with the initial diagnosis of Hodgkin’s lymphoma. Image a shows an example slice of a 3D ROI segmenting a lymphoma manifestation in the upper mediastinum. PET/CT (**b**,**c**) show this manifestation to be FDG-positive. Readers were required to include as much lymphoma manifestation as possible while keeping a distance of 1–2 pixels from the edge. A slight misalignment is also visible in (**b**), which we corrected when we copied the segmentation mask from the CT to the PET images.

Figure 1 shows an example of a 3D ROI.

### Defining the Deauville scores

To achieve reproducible results, we determined the final DS using the qPET approach^23^. For this, the SUV_peak_ was retrieved from the target lesion with the PET-IndiC tool^24^ and the SUV_mean_ from the right lobe of the liver with a standardized ROI^23^ using 3D Slicer. The relevant cutoff was between DS3 and DS4 and defined by a qPET value of 1.3^23^: lesions equal or above were classified as DS4-positive, lesions below as DS4-negative.

### Radiomic feature extraction

Radiomic features were extracted using PyRadiomics (Version 3.0)^25,26^, following the instructions of the Image Biomarker Standardisation Initiative (IBSI)^27^. The settings used for feature extraction can be found in the supplementary file S2a, the IBSI reporting guidelines and the checklist in the supplementary file S2b.

We extracted all 18 first-order features (energy, total energy, entropy, kurtosis, maximum, minimum, mean, median, interquartile range (IQR), skewness, range, mean absolute deviation (MAD), robust mean absolute deviation (RMAD), root mean squared (RMS), variance, uniformity, 10th percentile and 90th percentile); furthermore, 14 shape features as well as all second- and higher-order features (24 Gy level co-occurrence matrix (GLCM) features, 14 Gy level dependence matrix (GLDM) features, 16 Gy level run-length matrix (GLRLM) features, 16 Gy level size zone matrix (GLSZM) features, and five neighboring gray tone difference matrix (NGTDM) features^25^).

### Statistical analysis

The statistical analysis involved several steps. We tested all features scanner-wise for their diagnostic performance to evaluate if and which similarities exist between both datasets (single feature diagnostic performance). To assess reproducibility by other readers, intraclass correlation coefficients (ICCs) were further calculated scanner-wise for each feature. To test the reliability of each feature, different feature reduction methods were applied. Statistical analysis was performed using R (version 4.2.1, R Foundation for Statistical Computing)^28^. A p-value < 0.05 was generally considered to indicate statistical significance. If not otherwise stated, the reading by S.N.N. was considered.

#### Single feature diagnostic performance

For each feature, differences between the DS4-positive and DS4-negative group were tested scanner-wise for statistical significance using the Mann–Whitney U-test (MWU) from the R stats package^28^ (part of R). We further determined the diagnostic performance of each feature to classify a lesion as DS4-positive or DS4-negative with the receiver operating characteristic (ROC) curve analysis using the pROC package^29^ (Version 1.18.0). The resulting areas under the curve (AUCs) were rated as follows: 0.70–0.80 acceptable, 0.80–0.90 excellent, 0.90–1.00 outstanding diagnostic performance^30^.

#### Interreader agreement

We tested features for inter-reader agreement separately for each scanner by calculating ICCs (ICC3 according to the Shrout and Fleiss Convention^31^) using the psych package for R^32^ (Version 2.2.5). For this, we considered the readings from S.N.N. and L.J.J..

#### Feature reduction

We applied different methods to reduce the number of features in both datasets separately by dropping features with a correlation of more than 95% using the Hmisc package for R^33^ (Version 4.7.1), applying the minimum redundancy maximum relevance method from the praznik package for R^34^ (Version 11.0.0) selecting 20 (mrmr20) and 10 (mrmr10) features and with the recursive feature elimination (rfe) from the caret package for R^35^ (Version 6.0.93).

### Ethics approval and consent to participate

The study was conducted according to the guidelines of the Declaration of Helsinki, and approved by the Institutional Review Board (or Ethics Committee) of Charité Berlin (protocol code [EA1/104/19] and date of approval [5-14-2019]). Informed consent was obtained from all subjects involved in the study.

## Results

### Single feature diagnostic performance

Considering the results of the MWU test, 41 features from the dataset of scanner A and eight from the dataset of scanner B showed a significant difference between DS4-positive and DS4-negative lesions. Four of these features overlapped: mean, median, RMS, and 90th percentile.

**Figure 2:**
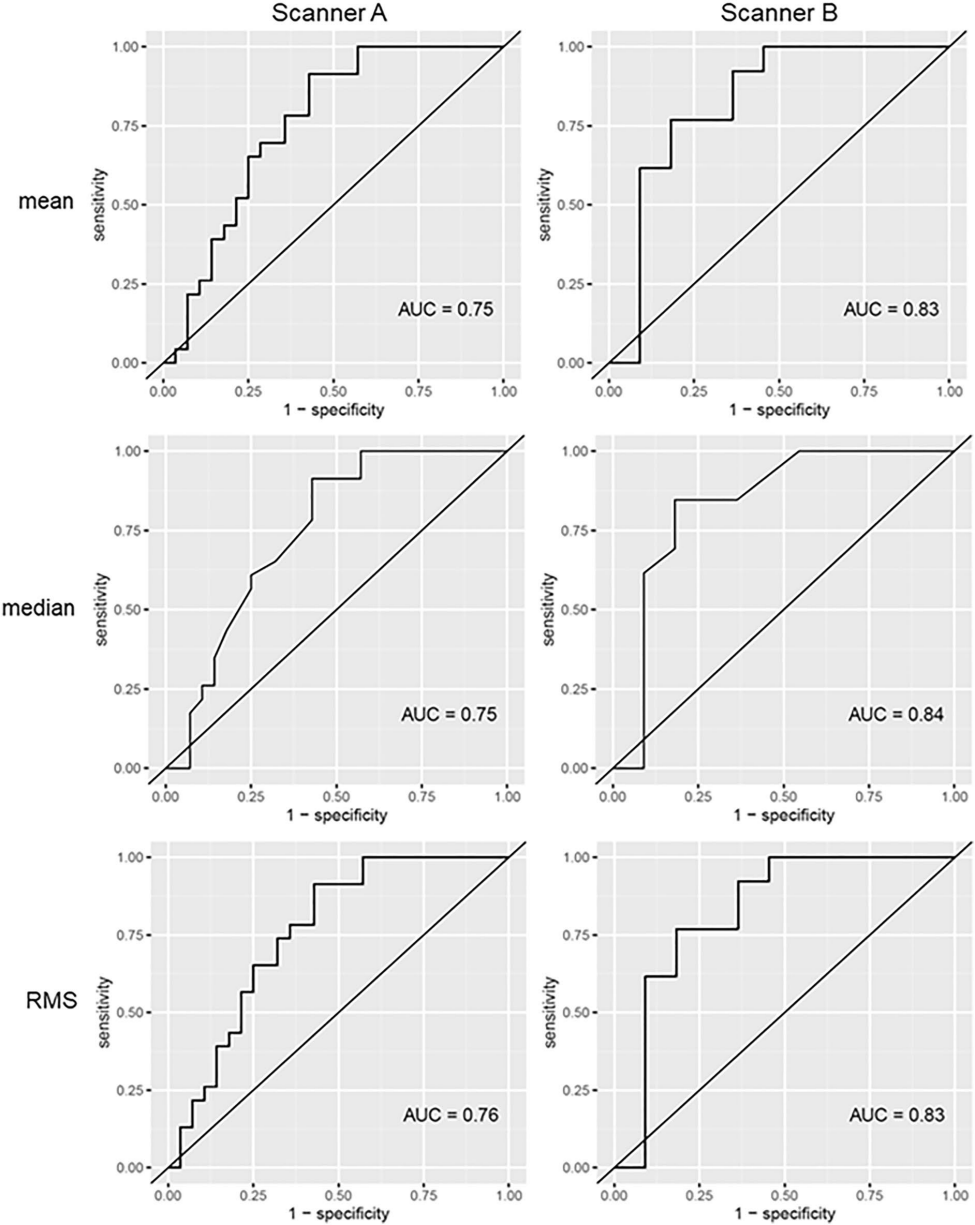
ROC curves of the three first-order features mean, median, and root mean squared (RMS), that concordantly distinguished Deauville score 4-positive and -negative lymphoma manifestations on both scanners. Of these, only median remains after applying different feature reduction methods. The left row shows the AUCs of Scanner A, and the right row the AUCs of Scanner B.

Considering the ROC analysis, 32 features from the dataset of scanner A and 19 from the dataset of scanner B showed an AUC of at least 0.7. Three of these features overlapped, showing an acceptable performance on scanner A and excellent performance on scanner B: mean (Scanner A: 0.75, B: 0.83), median (Scanner A: 0.75, B: 0.84), and RMS (Scanner A: 0.76, B: 0.83). ROC curves of these features are presented in Fig. 2. Table 4 offers a summary. Supplementary Tables S3 and S4 provide the full results of the MWU test (S3a,b) and ROC analysis (S4a,b) for scanners A and B.

**Table 4:** Overlapping features differentiating DS4-positive and DS4-negative lymphoma manifestations on both scanners.

	Scanner A				Scanner B			
	AUC	95% CI	Sensitivity	Specificity	AUC	95% CI	Sensitivity	Specificity
Mean	0.75	0.62–0.89	0.91	0.57	0.83	0.62–0.89	0.77	0.82
Median	0.75	0.61–0.88	0.91	0.57	0.84	0.61–0.88	0.85	0.82
RMS	0.76	0.63–0.89	0.91	0.57	0.83	0.63–0.89	0.77	0.82

Summary of the ROC analysis for scanner A and B. Only features with p < 0.05 in the Mann-Whitney-U-test and diagnostic performance > 0.70 on both scanners are shown. Only median remains after applying different feature reduction methods. Complete results are given in the supplementary Tables S3 and S4.

*RMS* root mean squared, *AUC* area under the curve, *95% CI* 95% confidence interval.

### Interreader agreement

Of all features from the dataset of scanner A, 25 showed an ICC below 0.8. Considering the dataset of scanner B, this accounted for 26 features. 21 features overlapped. A full list of features with an ICC below 0.8 is given in the supplementary Table S5.

### Feature reduction

Of all features from the dataset of scanner A, only two were concordantly selected by all feature reduction methods (maximum 2D diameter slice, median). On scanner B, only flatness and median were selected by all methods. The results of all feature reduction methods are provided in the supplementary Tables S6a (Scanner A) and S6b (Scanner B).

## Discussion

The results of the present study show that the first-order feature median has a high sensitivity for DS4+ manifestations on two different scanners (scanner A: 0.91, scanner B: 0.85). This feature could be easily applied on NECT images to estimate relevant metabolic activity when a PET scan is unavailable. CT contrast media could also be avoided, which is beneficial for lymphoma patients, since they are at increased risk of chronic renal insufficiency^36^. Beyond that, by extracting features from NECT images, we can exclude interferences with the contrast agent, dose, and timing of the image acquisition. Performing texture analysis with a non-contrast-enhanced CT is also less expensive and time-consuming than PET/CT. It would further limit radiation dose and is accessible country-wide^9^. Ganeshan et al. also attempted to extract prognostic information from non-contrast-enhanced CT scans of patients with Hodgkin’s lymphoma and aggressive Non-Hodgkin lymphoma complementary to interim FDG-PET/CT. They identified kurtosis associated with shorter progression-free survival of lymphoma patients, with analysis limited to first-order features^14^. Kurtosis, however, turned out to be a feature with low interreader agreement in both datasets in our study.

Reproducibility of radiomics is a general concern, restraining the implementation of radiomic signatures into clinical routine^37^. Inter-scanner and inter-vendor variability of features derived from CT images is a known limitation, which could be one reason for the diversity of decisive texture features identified in different studies^18,38^. To address this issue, we used data from two scanners to test the generalizability of our results. The first-order feature median derived from NECT concordantly had a high sensitivity for DS4+ manifestations on both scanners, indicating cross-scanner applicability.

Interestingly, even in NECT images, the feature median that ultimately describes the density of a lymphoma manifestation differentiated between DS4-positive and DS4-negative lesions, thereby showing constantly higher values in DS4-positive manifestations (numerical results of the radiomics analysis are provided in the supplementary Table S3; S3a for scanner A and S3b for scanner B). In their approach, Giesel et al. related lymph node density in Hounsfield units in NECT to malignancy in a broad PET/CT study investigating various malignant entities (lung cancer, malignant melanoma, prostate cancer, gastroenteropancreatic neuroendocrine tumors) using different PET-tracers (^18^F-FDG, ^68^Ga-DOTATOC, ^68^Ga-labeled prostate-specific membrane antigen), but without including patients with Hodgkin's disease^39^. They found that CT density correlated with ^18^F-FDG uptake, ^68^Ga-DOTATOC uptake, and ^68^Ga-PSMA uptake and suggested a Hounsfield scale to differentiate benign from malignant lymph nodes. Shao et al. investigated a lymph node/aorta density ratio in patients with non-small cell lung cancer undergoing preoperative ^18^F-FDG-PET/CT, pointing out a correlation between lymph node metastases and lymph node density^40^. Flechsig et al. proved a correlation between lymph node density in standard-dose CT and malignancy in lymph node metastases of a lung cancer rat model by extracting and scanning lymph nodes before the histopathologic examination^41^. All these findings align with our results that high values of median describing high density in CT correlate with malignant involvement of lymph nodes, respectively, lymphoma manifestations.

Our study has some limitations. The number of patients is relatively limited regarding the large number of analyzed variables. Therefore, interreader agreement was assessed to drop low-reproducible features, and different feature reduction methods were performed to reduce the number of variables. It would also have been desirable to obtain data from a more consistent patient population with a consistent therapy regimen and identical time points of PET/CT. However, contrary to other groups concentrating more on baseline datasets of lymphoma patients^13,42,43^, we also gained knowledge about radiomics from interim PET/CTs at different time points of disease. Our results should be verified in larger, more consistent patient populations examined on CT scanners from additional vendors to affirm median as a robust feature across scanners and should be validated externally according to Shahzadi^44^ supporting clinical applicability.

The first-order texture feature median describing lesion density derived from NECT concordantly has a high sensitivity for DS4+ Hodgkin manifestations on two different scanners. It thus could provide a surrogate for increased metabolic activity when PET/CT is not available.

## Supplementary Information

Supplementary Information.

## Data Availability

The datasets generated and/or analyzed during the current study are not publicly available due to an IRB decision which was made in the interest of ensuring patient confidentiality but are available from the corresponding author on reasonable request.

## Abbreviations

AUC: Area under the curve
CMR: Complete metabolic remission
CT: Computed tomography
DS: Deauville score
FDG: ^18^F-Fluorodeoxyglucose
FOV: Field of view
GLCM: Gray level co-occurrence matrix
GLDM: Gray level dependence matrix
GLRLM: Gray-level run-length matrix
GLSZM: Gray-level size zone matrix
IBSI: Image Biomarker Standardisation Initiative
ICC: Intraclass correlation coefficient
IQR: Interquartile range
kVp: Peak kilovoltage
MAD: Mean absolute deviation
MRMR10: Minimum redundancy maximum relevance with selection of 10 features
MRMR20: Minimum redundancy maximum relevance with selection of 20 features
MWU: Mann–Whitney U-test
NECT: Non-enhanced CT
NGTDM: Neighboring gray tone difference matrix
NPV: Negative predictive value
p: Level of significance
PET: Positron emission tomography
PPV: Positive predictive value
RFE: Recursive feature eliminiation
RMAD: Robust mean absolute deviation
RMS: Root mean squared
ROC: Receiver operating characteristic
ROI: Region of interest
SUV: Standardized uptake value
SUV_max_: Maximum SUV
SUV_mean_: Mean SUV
SUV_peak_: Peak SUV
