# Supplementary material for: CT radiomics to predict Deauville score 4 positive and negative Hodgkin lymphoma manifestations
Source: Sci Rep. 2022 Nov 21;12:20008. doi: 10.1038/s41598-022-24227-0 (PMC9678888; doi:10.1038/s41598-022-24227-0)
Supplement: Supplementary file 1 — Supplementary Information. [file 41598_2022_24227_MOESM1_ESM.zip › supplementary_information_legend.docx]

Supplementary information

File S1a Treatment of patients from Scanner A excel file

File S1b Treatment of patients from Scanner B excel file

File S2a PyRadiomics settings yaml file

File S2b IBSI reporting guidelines and checklist PDF file

Table S3a MWU test results for scanner A excel file

Table S3b MWU test results for scanner B excel file

Table S4a ROC analysis scanner A excel file

Table S4b ROC analysis scanner B excel file

Table S5 Features with an ICC below 0.8 excel file

Table S6a Feature reduction Scanner A excel file

Table S6b Feature reduction Scanner B excel file
